# Older portuguese and mexican adults and sexual well-being? A cross-cultural qualitative study

**DOI:** 10.1192/j.eurpsy.2021.1969

**Published:** 2021-08-13

**Authors:** S. Von Humboldt, N. Mendoza-Ruvalcaba, G. Low, I. Leal

**Affiliations:** 1 William James Center For Research, ISPA-Instituto Universitário, Lisbon, Portugal; 2 Division Of Health Sciences, University of Guadalajara CUTONALA, Guadalajara, Mexico; 3 Faculty Of Nursing, University of Alberta, Edmonton, Canada

**Keywords:** Mexican, older adults, sexual well-being, Portuguese

## Abstract

**Introduction:**

A cross-cultural qualitative study about older portuguese and mexican adults and sexual well-being.

**Objectives:**

Sexual well-being (SWB) refers to the subjective emotional and cognitive evaluation of the quality of the individual's sexuality, it plays a relevant role in quality of life and health promotion on old age and has cross-cultural implications. The aim of this study is to analyse comparatively the perspectives of older adults on their SWB in Portugal and Mexico.

**Methods:**

Data were collected from 86 Portuguese and 80 Mexican community-dwelling participants aged 65 years and older, using a semi-structured interview protocol. Older adults were inquired about their perceptions on what contributes to their sexual well-being. Socio-demographic data were also enquired. Content analysis was used to identify key themes.

**Results:**

Outcomes indicated eight themes: eroticism, supportive relationship, positive self-concept, health and self-care, romance, active life, tenderness and care, and no pain and no pregnancy restrictions, for both samples. Eroticism was the most frequent theme reported by Portuguese participants (31.4%) and health and self-care were the most frequent theme reported by Mexican participants (26.5%).

**Conclusions:**

The empirical results of this study indicated that SWB is strongly influenced by socio-cultural and psychosocial values. This cross-cultural comparison between Portugal and Mexico contributes to understand this concept in old age with different perspectives and place a scenario for future culture-adapted interventions and comprehensive policies.

**Disclosure:**

No significant relationships.

